# A partial *MECP2* duplication in a mildly affected adult male: a putative role for the 3' untranslated region in the *MECP2* duplication phenotype

**DOI:** 10.1186/1471-2350-13-71

**Published:** 2012-08-10

**Authors:** Neil A Hanchard, Claudia MB Carvalho, Patricia Bader, Aaron Thome, Lisa Omo-Griffith, Daniela del Gaudio, Davut Pehlivan, Ping Fang, Christian P Schaaf, Melissa B Ramocki, James R Lupski, Sau Wai Cheung

**Affiliations:** 1 Department of Molecular and Human Genetics, Baylor College of Medicine, One Baylor Plaza, Houston, TX 77030, USA; 2 Northeast Indiana Genetic Counseling Center, 11143 Parkview Plaza Drive, Suite 311 Fort Wayne, IN, USA; 3 Department of Pediatrics, Baylor College of Medicine, One Baylor Plaza, Houston, TX, USA; 4 Texas Children's Hospital, Houston, TX, USA

**Keywords:** Rearrangement, CNV, 3′ UTR, X-linked intellectual disability, Epilepsy

## Abstract

**Background:**

Duplications of the X-linked *MECP2* gene are associated with moderate to severe intellectual disability, epilepsy, and neuropsychiatric illness in males, while triplications are associated with a more severe phenotype. Most carrier females show complete skewing of X-inactivation in peripheral blood and an apparent susceptibility to specific personality traits or neuropsychiatric symptoms.

**Methods:**

We describe the clinical phenotype of a pedigree segregating a duplication of *MECP2* found on clinical array comparative genomic hybridization. The position, size, and extent of the duplication were delineated in peripheral blood samples from affected individuals using multiplex ligation-dependent probe amplification and fluorescence *in situ* hybridization, as well as targeted high-resolution oligonucleotide microarray analysis and long-range PCR. The molecular consequences of the rearrangement were studied in lymphoblast cell lines using quantitative real-time PCR, reverse transcriptase PCR, and western blot analysis.

**Results:**

We observed a partial *MECP2* duplication in an adult male with epilepsy and mild neurocognitive impairment who was able to function independently; this phenotype has not previously been reported among males harboring gains in *MECP2* copy number. The same duplication was inherited by this individual’s daughter who was also affected with neurocognitive impairment and epilepsy and carried an additional copy-number variant. The duplicated segment involved all four exons of *MECP2*, but excluded almost the entire 3' untranslated region (UTR)*,* and the genomic rearrangement resulted in a *MECP2*-*TEX28* fusion gene mRNA transcript. Increased expression of *MECP2* and the resulting fusion gene were both confirmed; however, western blot analysis of lysates from lymphoblast cells demonstrated increased MeCP2 protein without evidence of a stable fusion gene protein product.

**Conclusion:**

The observations of a mildly affected adult male with a *MECP2* duplication and paternal transmission of this duplication are unique among reported cases with a duplication of *MECP2*. The clinical and molecular findings imply a minimal critical region for the full neurocognitive expression of the *MECP2* duplication syndrome, and suggest a role for the 3′ UTR in mitigating the severity of the disease phenotype.

## Background

The *MECP2* gene maps to chromosome Xq28 and encodes a methyl CpG binding protein that acts as a transcriptional repressor or activator for genes associated with nerve cell function [1]. Duplications of *MECP2* have been described as the cause of Lubs syndrome (OMIM #300260), an X-linked recessive disorder in which affected males manifest a variety of moderate to severe neurological and cognitive phenotypes, including hypotonia, delayed or absent speech, intellectual disabilities, epilepsy, late-onset spasticity, as well as feeding difficulties and recurrent respiratory infections [2-7]. Most males have a reduced life-span, with death occurring within the first three decades of life; although older surviving individuals with severe impairment have been reported [8,9].

Most reported *MECP2* duplications are inherited from unaffected or mildly affected carrier mothers, with fewer arising *de novo*; as yet there have been no reported instances of paternal inheritance [4,10,11]. Carrier mothers typically have normal intelligence and demonstrate near 100% inactivation of the duplication-bearing X chromosome in DNA isolated from peripheral blood, but on more detailed testing most also have an apparent susceptibility to specific personality traits or neuropsychiatric concerns such as depression and anxiety [10,12]. Affected females with X:autosome translocations can have a phenotype that is similar to that seen in affected males; more typically, however, they have partially skewed X chromosome inactivation patterns with milder clinical features than affected males that include short stature, intellectual disability, body asymmetry and epilepsy [9,13-15].

Genotype-phenotype correlation studies suggest that the minimal duplicated region required to recapitulate the *MECP2* duplication phenotype includes the entire *MECP2* coding sequence and the neighboring *IRAK1* gene, and that *MECP2* is the primary dosage-sensitive gene mediating neurological outcomes [4,11,16,17]. This contention is further supported by a highly penetrant and more severe neurological phenotype observed with triplications of *MECP2*[12]. The molecular mechanisms modulating *MECP2* expression are complex and include a variety of *cis*-acting regulatory units, micro-mRNAs, and alternate transcripts [18]. The relative contributions and molecular interactions of these regulatory motifs, however, have yet to be fully elucidated, making the functional regulation of *MECP2* expression a subject of intense study and interest.

Here we provide extensive molecular characterization of a *MECP2* duplication occurring in a mildly affected adult male and subsequently transmitted to his affected daughter. The uniquely small size of the duplication, together with the unusual molecular and clinical attributes of the affected family, provided the opportunity to potentially gain functional insights into the neurocognitive phenotype of *MECP2* duplication syndrome.

## Results

### Neurocognitive abnormalities in a family segregating two copy number variants (CNVs)

#### Consultand

The consultand was a Caucasian female who presented at age three years and 10 months, after mild early delays in fine and gross motor development, with loss of speech and significant behavioral problems including attention deficit hyperactivity disorder (ADHD) and aggression. She also had hyperacusis, sensitivity to textures and poor eye contact. Formal autism evaluation using the Gilliam Autism Rating scales (GARS) was consistent with a diagnosis of pervasive developmental disorder not-otherwise-specified (PDD-NOS), and she was noted to also have problems with cognitive processing. She subsequently developed focal onset seizures and was diagnosed with epilepsy. She was proportionately large for her age (height between 75^th^ - 90^th^ percentiles, weight and head circumference at the 97^th^ and 98^th^ percentiles respectively) with mild dysmorphic features (smooth philtrum, double ear crus, over-folded helix, bilateral 5^th^ finger clinodactyly, talus rotation). When seen at age six years, her seizures were medically refractory, and she had a limited vocabulary with difficulties in speech articulation and required help with dressing and undressing.

#### Father

The 34-year-old father of the consultand related problems with speech and cognition in childhood that necessitated special education and speech therapy. He completed twelve years of school, and recalled being referred to as learning disabled. He was unable to read or to write. Specific developmental milestones were unavailable, but he did not recall a history of developmental delay. He experienced enuresis until age 18 years and epilepsy with generalized tonic-clonic seizures that began in infancy and continued until pre-adolescence. He did not endorse a history of childhood hypotonia, spasticity, ambulation problems, hospitalization, or frequent or recurrent infections. He was otherwise healthy and on no regular medications. He described himself as being moody and depressed, and he responded positively to all questions on a standard Mood Disorder Questionnaire [19]. On exam, his head circumference was normal, and he was non-dysmorphic apart from short palpebral fissures (more than four standard deviations below normal). He had a normal gait. He was conversant and friendly, but his cognitive processing appeared slow. He described living an itinerant lifestyle with intermittent employment as a day worker, and he gave no formal home address. Formal neuropsychiatric testing was declined, and he has not been available for further medical evaluation.

#### Pertinent family history

**Figure 1:**
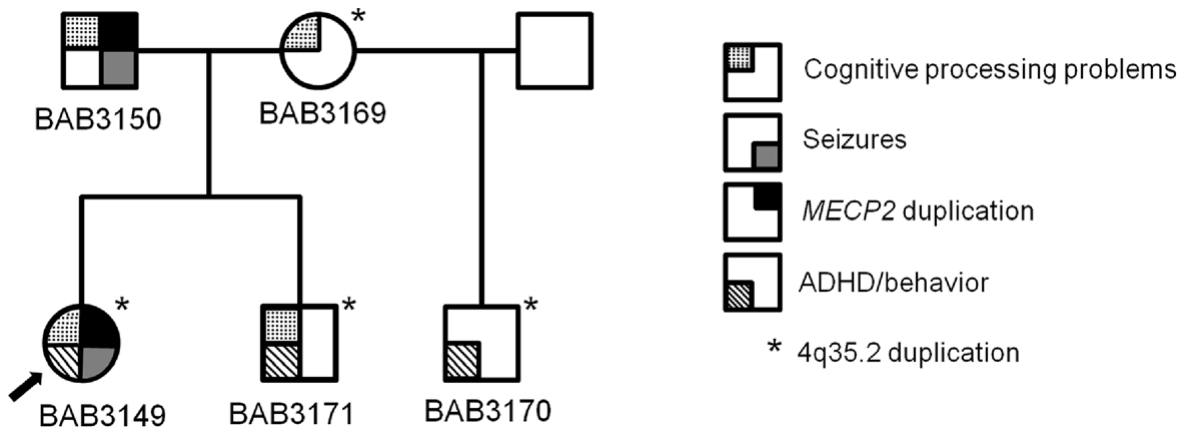
**Pedigree of affected family.** The pedigree shows segregation of two copy number variants (CNVs) and neurocognitive diagnoses in the family.

The remainder of the pedigree (Figure 1) was significant for a 4-year-old brother of the consultand with somnambulism and other sleep-related complex behaviors, ADHD, and Autism Spectrum Disorder (ASD). The consultand’s mother related difficulty maintaining employment as a result of problems with cognitive processing and executive functioning, for which she received special education during high school. The mother was otherwise healthy. In addition, a maternal half brother was diagnosed with sleep problems and ADHD.

### Partial duplications of *MECP2*

Microarray-based comparative genomic hybridization (array CGH) on DNA isolated from peripheral blood demonstrated two abnormalities in the consultand: a gain on chromosome 4q35.2 encompassing the *MTNR1A* and *FAT1* genes, sized between 194 and 296 kb, which was also present in the mother and both affected male siblings; and a gain on chromosome Xq28 estimated at approximately 115 kb involving the *MECP2* gene (Additional file 1). Array CGH of both parents and the consultand’s male half- and full- siblings indicated that the Xq28 gain in the consultand was paternally inherited and not present in either of the siblings (Figure 1). Fluorescence *in situ* hybridization (FISH) studies confirmed an interstitial duplication of Xq28, and multiplex ligation-dependent probe amplification (MLPA) confirmed that all four exons of *MECP2* were duplicated in both the consultand and her father (Additional file 2). X-inactivation studies (XCI) performed using the consultand’s DNA showed a moderately skewed pattern (83/17) in peripheral blood cells, with preferential inactivation of the paternally-inherited X-chromosome.

**Figure 2:**
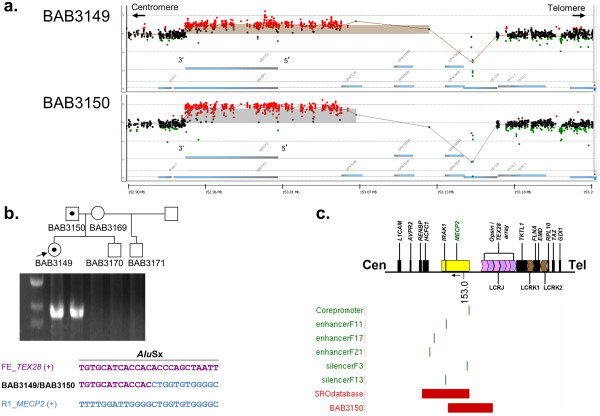
**Molecular characterization of the duplication involving *MECP2. *a**. High-resolution oligonucleotide array CGH results for the consultand (BAB3149) and father (BAB3150) showing a partial duplication of *MECP2*; **b**. PCR assay and sequencing result for the breakpoint junction of the duplication flanked by primers 3149R1 + FE. The junctional fragment was observed exclusively in those wells corresponding to consultand (BAB3149) and father (BAB3150); **c**. Smallest region of overlap (SRO) surmised from a cohort of 30 patients with *MECP2* duplication syndrome [11] encompassing *HCFC1*, *IRAK1*, *MECP2* as well as all known *MECP2 cis*-regulatory regions (shown in green) [24], compared with the partial duplication observed in the family (illustrated by BAB3150), which does not include the entire pre-defined SRO.

Further analysis using a high-resolution oligonucleotide microarray spanning the *MECP2* locus narrowed down the breakpoint junctions to a downstream breakpoint just distal (3′) of the gene and an upstream breakpoint more than 100 kb proximal (5′) of the gene (Figure 2a). A specific breakpoint junction product was obtained by PCR of DNA extracted from peripheral blood and confirmed in DNA from transformed lymphoblast cell lines in those samples that carried the duplication (i.e. consultand and father) (Figure 2b). Sanger dideoxy sequencing of the PCR product revealed no microhomology at the junction, suggesting Non-Homologous End Joining (NHEJ) as the mechanism for formation [11]. The proximal (centromeric) breakpoint mapped within exon 4 of *MECP2*, sparing almost the entire 3' UTR including three of the four postulated alternative polyadenylation sites [20]. The distal (telomeric) breakpoint mapped to an intron in one of two identical upstream pseudogenes of *TEX28*, implying that the full size of the duplication was 184.6 or 222.4 kb.

### Genomic context of the *MECP2* rearrangement

**Figure 3:**
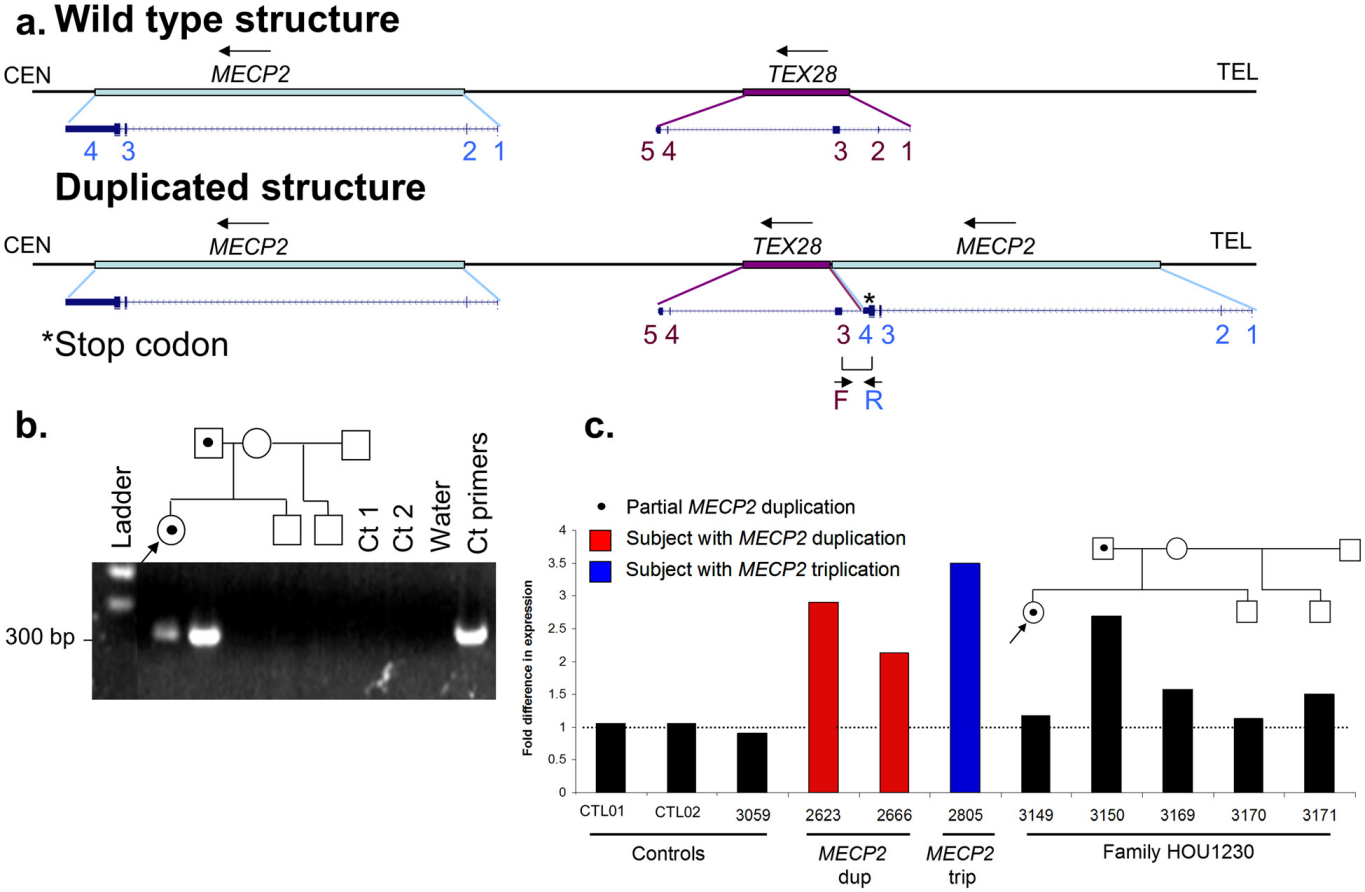
**Structural depiction and functional assay of partial *MECP2 *duplication. a**. Genomic structure for consultand (BAB3149) and father (BAB3150) surmised using array CGH and breakpoint sequencing analysis. Both wild type (reference) and post-duplication structures are shown for comparison; **b**. PCR assay and sequencing results for the *MECP2*-*TEX28* fusion gene junction flanked by primers Exon4_R + TEXon2_F3; **c**. Relative *MECP2A* mRNA assay for affected family (HOU1230). Control samples: CTL02 and 3059 are males carrying one copy of *MECP2*; CTL01 is a female carrying one copy of *MECP2*; 2623 and 2666 are male patients carrying two copies of *MECP2 *[11]; 2805 is a male patient carrying three copies of *MECP2 *[12]. Relative fold of mRNA expression changes were calculated using the comparative threshold cycle method (ddCT).

We investigated whether the juxtaposition of the duplicated *MECP2* gene adjacent to *TEX28* (Figure 3a) produced a fusion gene that might be relevant to the familial phenotypes. PCR was performed using cDNA, with primers designed at the ends of exon three of *TEX28* and exon four of *MECP2*. We identified a genomic rearrangement-associated novel fusion gene in the father and consultand daughter exclusively, using RNA extracted from lymphoblast cell lines. This result was consistent with partial expression of the *MECP2* duplicated product in the peripheral blood of the consultand, despite her having moderately skewed X-inactivation. Sequencing of the resulting PCR product (Figure 3b) confirmed the gene fusion.

We next sought to determine whether the insertion of a partial copy of *MECP2* in a new genomic context would still be associated with increased *MECP2* transcription. We used the transcriptional expression of the *MECP2A* isoform (also known as *MECP2-e2* isoform) as a surrogate marker of the normal *MECP2* copy, as this isoform is known to be expressed in lymphoblasts [21]. We found that relative to control lymphoblast cell lines, the father demonstrated markedly increased *MECP2A* transcription, consistent with the levels of transcription typically observed in patients carrying *MECP2* duplication and triplication (Figure 3c). This was not observed for any other family members including the consultand. The latter observation may reflect the fact that the clonally obtained lymphoblast cell lines of the female consultand demonstrated more extensive skewing of X-inactivation (96/4, versus 83/17 in peripheral blood) as a consequence of the cloning passage [22]; the lack of appreciable increase in expression in the consultand is therefore considered to be equivocal.

**Figure 4:**
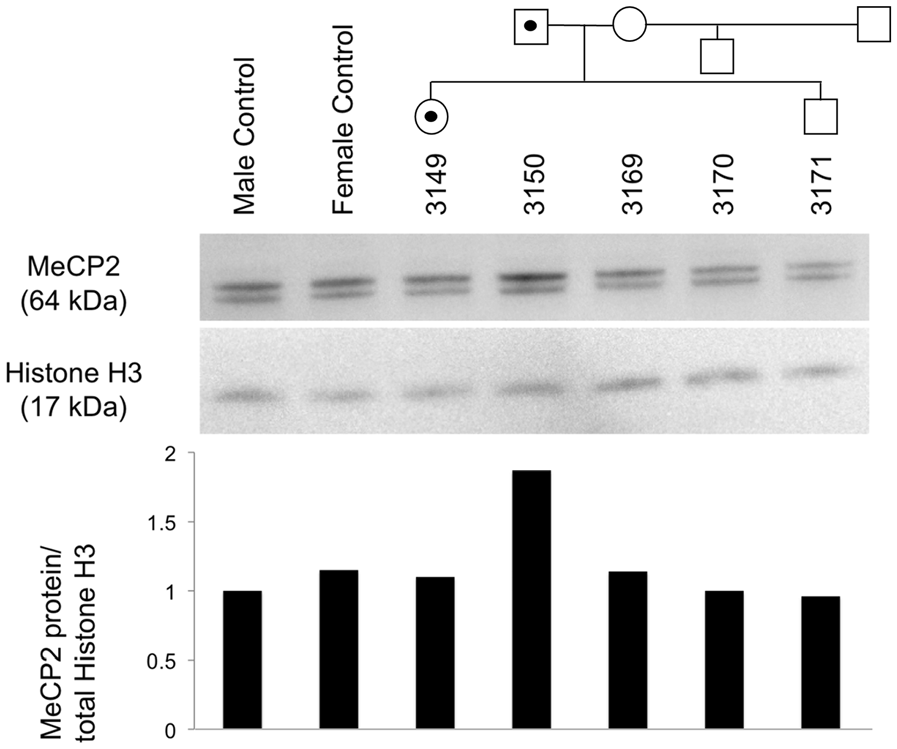
**Comparison of MeCP2 protein levels in pedigree members.** MeCP2 protein levels are normalized to histone H3. Individuals 3149 (consultand), 3169 (mother), 3170 (maternal half-brother) and 3171 (brother) have MeCP2 protein levels similar to normal control individuals (female control – CTL01, male control – CTL02); lymphoblasts of individual 3150 (father) produced 1.9-fold the amount of MeCP2 protein observed in cells from control individuals.

Having observed an apparent increase in *MECP2A* transcription in the consultand’s father, we then sought to determine whether the increased *MECP2* transcription would result in an increase in MeCP2 protein or MeCP2-TEX28 fusion protein. Proteins were extracted from immortalized lymphoblast cell lines of all available family members and a male and female control cell line. MeCP2 protein levels were then quantified and compared to total histone H3 levels used as loading control. Consistent with the results from the transcription experiment, lymphoblasts of the consultand’s father (BA3150) produced 1.9-fold the amount of MeCP2 protein than cells from control individuals, while the other family members (including the consultand (BA3149)) had MeCP2 protein levels similar to normal control individuals (Figure 4). Further, there was no evidence of an additional band in the consultand or her father, which would be expected if a viable fusion protein (MeCP2-TEX28) were formed.

## Discussion

A *MECP2* duplication, partial or complete, occurring in an adult male with minimal symptomatology has not, to the best of our knowledge, been previously reported; neither has paternal transmission of a *MECP2* duplication to an affected female offspring. This family is thus both novel and unique among reported patients with *MECP2* duplications. In the affected male, the partial duplication is associated with increased *MECP2* expression and MeCP2 protein production, and occurs in the context of a clinical presentation that is distinct from the ‘typical’ phenotype of adult males with *MECP2* duplication, who usually manifest a greater degree of neurological impairment. The key to this apparent phenotypic conundrum appears to lie with the breakpoints, size, and ultimate physical position of the duplicated segment, all of which are also unique among reported *MECP2* duplications.

We previously reported a series of 30 male patients with *MECP2* duplications [11], in which the duplication sizes were between 250 kb and 2.6 MB - larger than the 184 or 222 kb size estimated for the consultand’s father. A duplication of similar small size has been reported in an apparently non-dysmorphic female referred for intellectual disability [13], but this duplication was translocated to chromosome 10, and no functional information was reported. The reported smallest region of overlap (SRO) in our series was approximately 140 kb and included both *MECP2* and the neighboring *IRAK1* gene [4,11]. By comparison, in our present case, the genomic breakpoints are more telomeric (Figure 2c), ostensibly leaving *IRAK1* dosage unperturbed. Increased *IRAK1* gene dosage has been associated with a history of recurrent infections in some *MECP2* duplication patients. This assertion would be consistent with the father’s negative history of recurrent respiratory or other infection; however, given the variable expressivity of the infection phenotype, additional studies are needed to determine whether increased *IRAK1* dosage is necessary and/or sufficient to manifest recurrent infections in *MECP2* duplication patients.

We speculate that the father’s neurological history, or lack thereof - an affected male with speech delay and epilepsy but without the hypotonia, spasticity, or degree of cognitive and language impairment typically observed in boys with *MECP2* duplications – may be the consequence of the specific rearrangement breakpoints within the *MECP2* gene. We detected increased expression of the *MECP2A* mRNA isoforms in his blood and consistently increased MeCP2 protein on western blot. There is evidence from the literature that the *MECP2A* isoform may not be as biologically relevant in brain as isoform *MECP2B*[21]. In fact, the ‘long’ *MECP2* transcript of 10.2 kb, which is the predominant transcript in the brain, appears to correspond to *MECP2B* and is present in full only once in this present rearrangement. The duplicated region is predicted to produce only the ‘short’ *MECP2* transcript of 1.8 kb, which has higher expression in non-neurological tissues such as heart and muscle [21]. Recent studies using a Cre-LoxP system that specifically disrupts the *MECP2A* isoform (also known as *MECP2-e2*) have demonstrated that specific loss of the short transcript does not result in a neurological phenotype in mice [23]. Alternatively (or in addition), it is possible that in the absence of *MECP2*-associated *cis*-regulatory elements [24] from the duplicated segment (Figure 2c), the regulation of the duplicated copy in brain may be different from that observed with the wild-type copy. These observations concur with mouse models of Rett syndrome in which disruption of the 3'UTR has been shown to alter MeCP2 protein stability and activity [25,26]. The genomic configuration of the rearrangement together with the clinical picture, lead us to speculate that the overall dosage effect of *MECP2* on neuronal activity may be mitigated by effects attributable to the unduplicated 3' UTR segment, resulting in fewer adverse clinical manifestations than are typically seen.

Lastly, although both the consultand and her father share the same duplicated segment, the consultand clearly had a more severe phenotype than her father. It is unusual to find a female with a duplication of *MECP2* who is more severely affected than a male family member, since, by definition; females cannot have more skewing than males. Although there exists the possibility of rare *cis*-acting modifiers of transcription and/or translation a more parsimonious explanation is of a ‘2^nd^ hit’ in which an additional genomic event (structural variant or point mutation) results in a more severe phenotype in the consultand than that expected from the father’s *MECP2* duplication [27-29]. The segregation of other neurocognitive phenotypes within the nuclear family gives further credence to this suggestion; the consultand’s mother and two maternal siblings demonstrated a range of neurocognitive and behavioral phenotypes that were similar to the consultand, but none gave a history of seizures. It may be that seizures and some of the neurocognitive defects segregate with the partial *MECP2* duplication, whilst autistic features, other additional cognitive defects, and sleep problems are the result of a 2^nd^ hit. As the immediate maternal family members all carried the 4q35 duplication, but *not* the *MECP2* duplication, the 4q35 duplication could represent the 2^nd^ genomic hit in this family. The 4q35 CNV is a small 100 kb region that encodes the melatonin receptor *MTNR1A* and the transcription factor *FAT1*. Neither of these genes are known to be dosage-sensitive or disease-causing and a preliminary enquiry of the DECIPHER database (The DECIPHER Consortium, http://decipher.sanger.ac.uk) indicates that larger encompassing duplications have been seen, without a clear clinical phenotype and occasionally inherited from a reportedly normal parent. The segregation of the phenotypes in this family, however, suggests that there may yet be a role for this region in neurocognition and sleep. This is a subject of ongoing study in our laboratory.

## Conclusion

Our findings lead us to suggest that the severe neurological and cognitive phenotypes typically observed in the *MECP2* duplication syndrome result from duplication of the entire *MECP2* coding *and cis*-regulatory elements. This is consistent with the observation that most, if not all, of the neurological phenotypes observed in the *MECP2* duplication syndrome can be recapitulated by duplication of *MECP2* alone in mice [16]. Tacitly, our findings imply that copy number gain of the ‘short’, as opposed to the ‘long’, *MECP2* transcript and/or disruption of specific 3' regulatory motifs may be associated with an overall milder neurocognitive phenotype, but one that remains susceptible to the development of epilepsy. Given that interpretation of the likely clinical consequences of intragenic duplications can often be challenging without specific breakpoint resolution of the rearrangement [30,31], our findings have potential prognostic implications for genetic counseling of males with intragenic duplications of *MECP2*. Further studies of the role of the 3′ UTR in modifying the *MECP2* duplication phenotype are required to validate the hypotheses generated from this unique family, but, if confirmed, this could become an avenue of fertile and important research going forward.

## Methods

### Samples

Initial samples from the consultand daughter (BAB3149) were received as a part of her clinical diagnostic evaluation; thereafter, written informed consent was procured for additional research testing on samples from the consultand (BAB3149) as well as the remainder of the nuclear family members (BAB3150 - father, BAB3169 - mother, BAB3170 - maternal-half brother, BAB3171- brother). All studies were performed with the approval of the Baylor College of Medicine Institutional Review Board.

### Clinical laboratory studies

Chromosomal microarray analysis, GTG-banded (G-bands obtained with trypsin and Giemsa) chromosome analysis, FISH, and MLPA analyses were performed at Baylor College of Medicine Medical Genetics Laboratories (http://www.bcm.edu/geneticlabs/) as described previously [4,32]. X-inactivation studies were based on the protocol described by Allen [32,33] with modifications as described previously [10].

### Duplication size and genome content

To determine the size, genomic extent and gene content for each rearrangement, we designed a tiling-path oligonucleotide microarray spanning 4.6 Mb across the *MECP2* region on Xq28. The custom 4x44k Agilent Technologies (Santa Clara, CA) microarray was designed using the Agilent e-array website (http://earray.chem.agilent.com/earray/). We selected 22,000 probes covering ChrX: 150,000,000-154,600,000 (NCBI build 36), including the *MECP2* gene, which represents an average distribution of 1 probe per 250 bp. Probe labeling and hybridization were performed as described previously [11].

### Quantitative real-time PCR

Total RNA was extracted from immortalized lymphoblast cell lines from all family members, three controls without copy number variation in the Xq28 region (CTL01, female control; CTL02, male control; 3059 male control), two individuals carrying *MECP2* duplications (BAB2623 and BAB2666)[11], and one individual carrying a *MECP2* triplication (BAB2805)[12]. RNA extraction was carried out on lymphoblasts using TRIzol reagent (Invitrogen Corporation, Carlsbad, CA); this was DNaseI treated and purified using the RNeasy mini kit according to the manufacturer’s protocol (Qiagen, Valencia, CA). cDNA was synthesized from 1 μg of RNA using qScript cDNA Super Mix (Quanta Biosciences, Gaithersburg, MD). Quantitative real-time PCR reactions were performed using TaqMan expression assay Hs00172845_m1 from Applied Biosystems (ABI; Life Technologies, Carlsbad, CA) that specifically detects *MECP2A* transcript. Experiments were carried out according to the manufacturer’s protocol. Four technical replicates were performed for each genomic DNA sample and normalized relative to *TBP* levels. Reactions were run on the ABI 7900HT fast system. Relative fold of mRNA expression changes were calculated using the comparative threshold cycle method (ddCT).

### PCR amplifications

Primers for long-range PCR were designed at the apparent boundaries, as denoted by transitions signifying a gain of the duplicated segment relative to the reference genome as inferred from the custom oligonucleotide microarray result. Long-range PCR was performed using TaKaRa LA Taq (Clontech, Mountain View, CA). Primers to obtain the breakpoint junction 3149R1: 5'AGAGAAGATGGATATGACCAGTGGC 3′ and’ FE: 5' GCCTCACCTACACTTCTCCTCCTG 3'. Primer 3149FW1: 5′ TCTACATGTGTGCCTCACT 3′ was used to walk across the 3149R1 + 3149FE PCR product. Detection of the transcript from the fusion gene junction was performed using cDNA from primers: Exon4_R:CCGTGACCGAGAGAGTTAGC and TEXon2_F3:ACTGGCCTTCTCCACTCTCA.

### Acid extraction of protein and western blot analysis

Proteins were isolated via acid extraction from confluent cultures of immortalized human lymphoblast cell lines of subjects BA3149, BA3150, BA3169, BA3170, BA3171, a male control individual, and a female control individual. Acid extraction of protein, western blotting, and Immunodetection with anti-MeCP2 antibody 0535, were performed according to our previously published protocol [10]. Immunodetection with antihistone H3 antibody (Millipore, Billerica, MA, #06-755, 1:100,000 dilution) was performed as a loading control. MeCP2 and histone H3 protein levels were quantified using ImageJ software (National Institutes of Health, Bethesda, MD) and the total MeCP2 protein level was normalized to the level of histone H3 protein detected for each subject.

## Competing interests

NH, CC, PF, DP, CS, JRL and SWC are based in the Department of Molecular and Human Genetics at Baylor College of Medicine, which offers genetic laboratory testing, including the use of arrays for genomic copy number analysis, and derives revenue from this activity. JRL is a consultant for Athena Diagnostics, has stock ownership in 23andMe and Ion Torrent Systems, and is a co-inventor on multiple United States and European patents for DNA diagnostics. None of the remaining authors have any competing interests to disclose.

## Authors’ contribution

nh collated the clinical information, aided in the conceptualization and design of the study, and wrote the manuscript; CC performed the confirmatory experiments, analyzed the data, and assisted in the conceptualization of the paper and the writing of the manuscript; AT assisted in the writing of the manuscript and the conceptualization of the study; PB and LOG evaluated and managed the clinical aspects of the case and contributed to the writing of the manuscript; DdG performed and interpreted the MLPA studies, DP assisted in the performance and analysis of confirmatory experiments; PF oversaw and analyzed the X-inactivation studies; CS performed the western blot analysis and contributed to the drafting of the manuscript. MB assisted in the design and conceptualization of the study, was responsible for the cell line establishment, and assisted in the writing of the manuscript; JRL was involved in the conceptualization, execution and analysis of the confirmatory experiments, and helped to draft the manuscript. SWC conceived the study, analyzed and interpreted the cytogenetic studies and array data, and helped draft the manuscript. All authors read and approved the manuscript for submission.

## Pre-publication history

The pre-publication history for this paper can be accessed here:

http://www.biomedcentral.com/1471-2350/13/71/prepub

## Supplementary Material

Additional file 1 **Clinical arrayCGH of *MECP2 *and adjacent regions in consultand.** Description: The array plot illustrates a gain in copy number (green dots) spanning *MECP2* and the adjacent 5′ region in the consultant (BAB3149). Black dots represent normal gene dosage (hybridization intensity) compared to sex-matched controls. Red dots represent oligos with log2 ratios less than −0.3 suggestive of a heterozygous loss**.** Click here for file

Additional file 2 **Clinical laboratory confirmation of *MECP2 *duplication.** Description: a. MLPA of *MECP2* in the consultand’s father (BAB3150) demonstrating increased dosage (blue peaks) compared with a normal male control (red peaks). Red asterisks indicate the duplicated *MECP2* exons, with MECP2_exon 01 (Start) and MECP2_exon04c (End) probes representing the first and the last duplicated probes in the coding region. Probes located in the proximal *L1CAM* gene and in the 3′ UTR of the *MECP2* gene (*MECP2* exon 04b) show normal dosage. b. Fluorescent in-situ Hybridization (FISH) using probes specific for the duplicated region and showing the duplication to be on the X chromosome. Click here for file
