# Supplementary figures and images for: A partial MECP2 duplication in a mildly affected adult male: a putative role for the 3' untranslated region in the MECP2 duplication phenotype
Source: BMC Med Genet. 2012 Aug 10;13:71. doi: 10.1186/1471-2350-13-71 (PMC3575261; doi:10.1186/1471-2350-13-71)

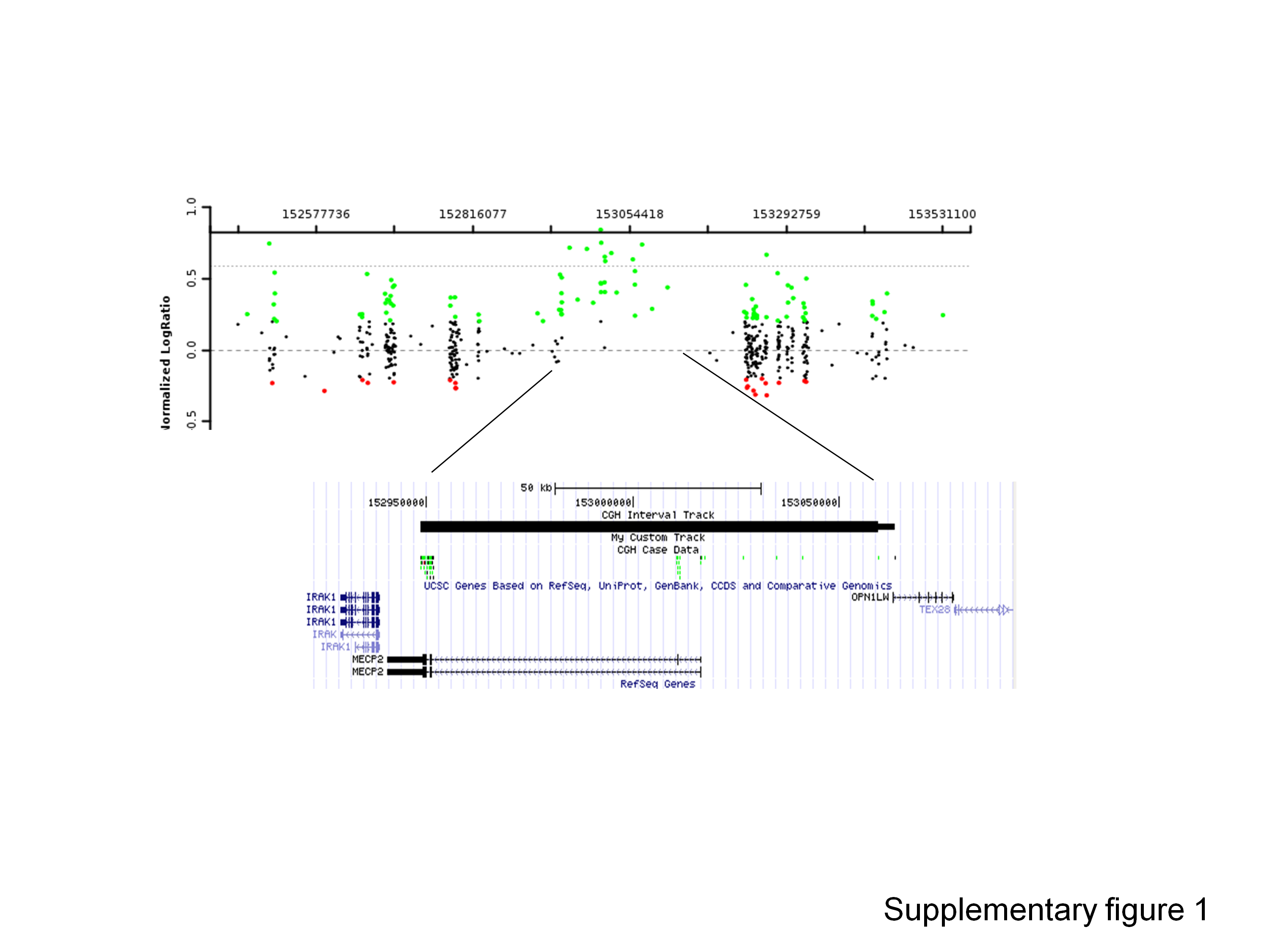

Supplement: Additional file 1 — Clinical arrayCGH of MECP2 and adjacent regions in consultand. Description: The array plot illustrates a gain in copy number (green dots) spanning MECP2 and the adjacent 5′ region in the consultant (BAB3149). Black dots represent normal gene dosage (hybridization intensity) compared to sex-matched controls. Red dots represent oligos with log2 ratios less than −0.3 suggestive of a heterozygous loss. [file 1471-2350-13-71-S1.tiff]

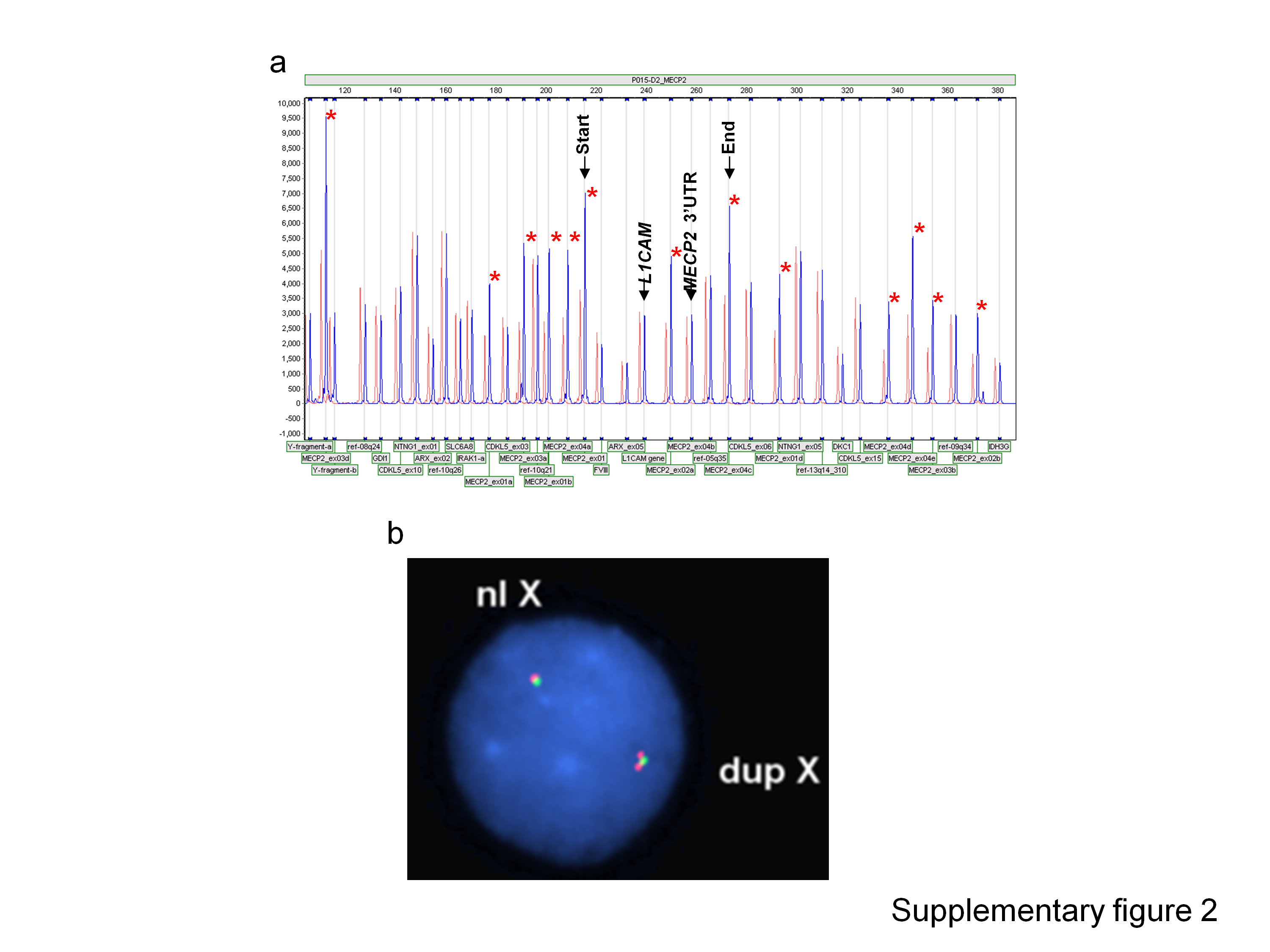

Supplement: Additional file 2 — Clinical laboratory confirmation of MECP2 duplication. Description: a. MLPA of MECP2 in the consultand’s father (BAB3150) demonstrating increased dosage (blue peaks) compared with a normal male control (red peaks). Red asterisks indicate the duplicated MECP2 exons, with MECP2_exon 01 (Start) and MECP2_exon04c (End) probes representing the first and the last duplicated probes in the coding region. Probes located in the proximal L1CAM gene and in the 3′ UTR of the MECP2 gene (MECP2 exon 04b) show normal dosage. b. Fluorescent in-situ Hybridization (FISH) using probes specific for the duplicated region and showing the duplication to be on the X chromosome. [file 1471-2350-13-71-S2.tiff]
